# DNA Methylation in Stroke. Update of Latest Advances

**DOI:** 10.1016/j.csbj.2017.12.001

**Published:** 2017-12-09

**Authors:** Jerzy Krupinski, Caty Carrera, Elena Muiño, Nuria Torres, Raid Al-Baradie, Natalia Cullell, Israel Fernandez-Cadenas

**Affiliations:** a Neurology Service, Hospital Universitari Mútua Terrassa, Terrasa, Barcelona, Spain; b School of Healthcare Science, Manchester Metropolitan University, Manchester, United Kingdom; c Neurovascular Research Laboratory, Institut de Recerca, Universitat Autònoma de Barcelona, Hospital Vall d'Hebron, Barcelona, Spain; d Stroke Pharmacogenomics and Genetics, Fundació Docència i Recerca Mutua Terrassa, Hospital Universitari Mútua de Terrassa, Terrassa, Barcelona, Spain; e Applied Medical Sciences College Majmaah University, Majmaah, Saudi Arabia; f Stroke Pharmacogenomics and Genetics, Sant Pau Institute of Research, Hospital de la Santa Creu i Sant Pau, Barcelona, Spain

**Keywords:** Stroke, Epigenetics, Genetics, EWAs, GWAs

## Abstract

Epigenetic modifications are hereditable and modifiable factors that do not alter the DNA sequence. These epigenetic factors include DNA methylation, acetylation of histones and non-coding RNAs. Epigenetic factors have mainly been associated with cancer but also with other diseases and conditions such as diabetes or obesity. In addition, epigenetic modifications could play an important role in cardiovascular diseases, including stroke. We review the latest advances in stroke epigenetics, focusing on DNA methylation studies and the future perspectives in this field.

## 1 Introduction

Stroke is the 2nd leading cause of death worldwide. It is also the leading cause of disability in adults, with 20% of stroke survivors needing help to walk and being dependent on others to perform daily tasks. Stroke survivors therefore require significant social and healthcare resources [1].

In the elderly population of 15 European countries during the year 2000, estimates showed 2,700,000 recurrent stroke cases, and 536,000 incident stroke cases per year [2]. The total number of deaths due to stroke in the total European Union (EU) members is estimated at 508,000/year. Given that age is one of most important risk factors for stroke, the aging of the world population implies a growing number of people at risk. An international comparison of stroke cost studies showed that 0.27% of domestic product was spent on stroke care by national health systems, and stroke care accounted for approx. 3% of total healthcare expenditure [3].

### 1.1 Stroke Recurrence

The risk of ischemic stroke recurrence after a first stroke is high, especially in the early stages, being around 6–12% within the first year of the initial stroke [4]. Moreover, stroke patients also have a high risk of developing other vascular diseases such as acute myocardial infarction and vascular death. Data suggest that within 10 years of having an ischemic stroke or Transient Ischemic Attack (TIA), around 60% of patients will die and 54% will experience a new vascular event.

### 1.2 Stroke Functional Outcome

The variability in functional status and neurological outcome after stroke can be influenced by many factors, including age, haemorrhagic transformation (HT), infarct size and location, or the efficiency of revascularisation (by thrombolytic drugs or mechanical thrombectomy) [5], [6]. Results from previous studies suggest that basal glucose, age, hypertension and arterial revascularisation success accounted for 33% of the variability in neurological outcome during the acute phase of stroke (at 24 h). However, 57% of the neurological variability remains unexplained. In addition, 25% of neurological outcome could be explained by common polymorphisms or single nucleotide polymorphisms (SNPs) [7].

### 1.3 Stroke Genetics

Stroke is influenced by genetic risk factors. These genetic risk factors can affect stroke occurrence, acute outcome, long-term outcome and vascular recurrence, among others. Interestingly, different molecular pathways modulate these processes, suggesting that different genetic risk factors influence these processes.

Different Genome Wide Association studies (GWAs) have been performed in ischemic stroke and different loci have been associated with the risk of suffering an ischemic stroke. Specifically, 3 genes have been associated with atherothrombotic stroke subtype (HDAC9, CDKN (locus 9p21) and TSPAN2), 2 genes with cardioembolic stroke subtype (PITX2 and ZFHX3), and 2 genes with young strokes (ABO and MMP12). Other GWAs analyses have found other genes and loci associated with stroke (PRKCH, NINJ and genetic locus 6p21.1), although further studies are needed to confirm those results [8], [9], [10], [11], [12], [13], [14], [15], [16], [17].

In relation to stroke recurrence several studies in Asian populations [18], [19] associations of *ANRIL* and *NINJ2* polymorphisms with vascular recurrence. Other studies in Caucasian populations found associations between *CRP* and *MGP* polymorphisms and recurrent stroke [4], [20]. However, other studies have not observed those associations [21].

Different polymorphisms in the genes MMP2, COX-2, GPII, and TP53 have been associated with post-stroke outcome [22], [23], [24], although these results have not been consistently replicated.

### 1.4 Epigenetics

The risk associated with the genetic background in stroke is in the order of 37.9% [25], [26]. However, the genetic risk associated with the variants found to date only account for 5–10% of that genetic risk [25]. Therefore, there are more genes and heritable risk factors associated with stroke that have not yet been discovered. One of these possible heritable changes could be associated with epigenetic modifications.

Epigenetic mechanisms are known to alter gene expression or cellular phenotype [27].

There are three major components of epigenetic modification: a) methylation, b) histone modifications and c) non-coding ribonucleic acid (RNA) interference. Both methylation and histone modifications join hands to provide a dynamic epigenetic code. Along with non-coding RNAs (ncRNAs) and certain interacting proteins, these modifications regulate the transcription process.

#### 1.4.1 Methylation

DNA methylation and modifications in histone proteins are the most intensively studied among the major epigenetic modifications. DNA methylation occurs when a methyl group is added to a cytosine nucleotide that precedes guanines (so-called CpG islands or CpG sites).

A CpG island may be defined as the DNA region of at least 500 base pairs with a CG content of > 55% [28]. Methylation of CpG islands is catalysed by a family of enzymes, the DNA methyl transferases (DNMTs). DNMT1 maintains cytosine methylation through mitotic and meiotic cell divisions. Methylation of a CpG island within gene promoters is commonly associated with repressed gene expression, as it impedes the binding of transcription factors.

#### 1.4.2 Other Epigenetic Modifications

Post-translational histone modifications, such as methylation and acetylation of lysine residues on histone tails, affect gene expression mainly by altering chromatin structure [27]. Acetylation is brought about by histone acetyltransferase (HATs) enzymes and deacetylation by histone deacetylases (HDACs) [29], [30].

Small non-coding RNAs (sncRNAs) are epigenetic elements (< 30 nucleotides) with a post-transcriptional biological function. The major components of the sncRNA family, the microRNAs (miRNAs), generally interact specifically with the 3′ untranslated region of a target mRNA to induce its cleavage and degradation, or via a translational repression of gene expression [31], [32].

Epigenetic mechanisms are known to alter gene expression [27]. However, other underlying mechanisms such as genetic variations could modify DNA CpG sites modifying the epigenetic regulation of genes [33]. Knowing these mechanisms could be important in finding new treatments for stroke and other cardiovascular diseases [34].

### 1.5 Epigenome-Wide Association Studies (EWAS). Technical Aspects of the Methylation Chips

Genome-wide association studies (GWAS) have been powerful tools in the identification of the most common genetic variants associated with a multitude of complex traits including common diseases. In contrast, the systematic assessment of epigenetic variation has lagged behind. Technological advances in high-throughput DNA analysis have facilitated the genome-wide examination of epigenetic modifications, primarily DNA methylation. Epigenome-wide association studies (EWAS) have provided systematic, large-scale association testing with disease phenotypes. The latest EWAS arrays (the Infinium EPIC HumanMethylation BeadChip (Illumina)) can detect the methylation levels of > 800,000 CpG sites across the genome.

Numerous diseases, exposures and lifestyle factors have been investigated by EWAS, with several significant associations now identified. However, much like the GWAS studies, EWAS are likely to require large international consortium-based approaches to reach the numbers of subjects, and statistical and scientific rigour, required for robust findings.

#### 1.5.1 Tissue-Specific Methylation

DNA methylation is strongly influenced by the tissue analysed and the environment. In fact, epigenetics is one of the metabolic factors that regulate the different expression pattern of the cells and tissues. Consequently, epigenetic studies should be performed in the key tissue for the disease or the condition. In addition, in the case of blood samples there are different cell types with different DNA methylation pattern. This should be taken into consideration before EWAs analysis in order to normalise the results.

Taking into consideration the role of genetics in the risk of stroke but also the outcome after a stroke, DNA methylation could be associated with the occurrence of stroke, with stroke recurrence and with functional outcome after stroke.

## 2 DNA Methylation in Stroke

### 2.1 Methods

**Fig. 1:**
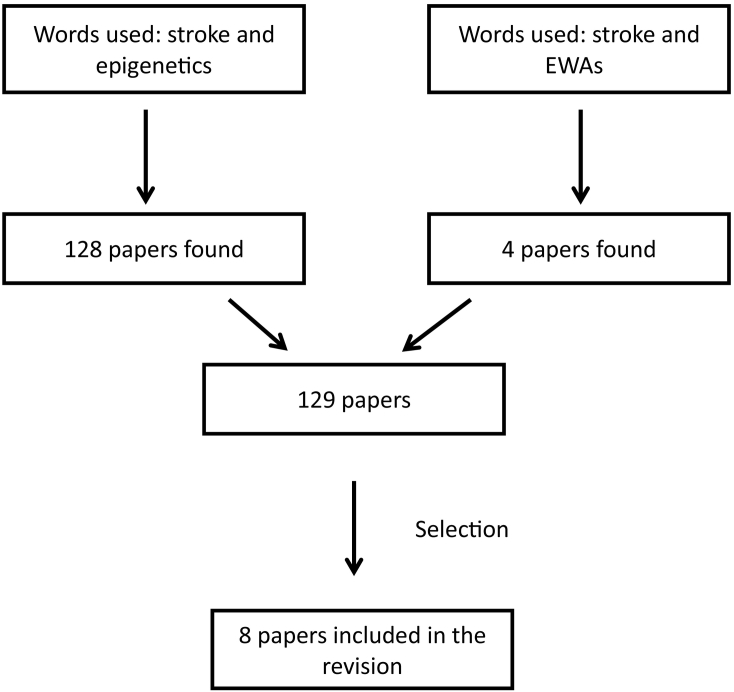
Work flow showing the selection of the studies included in the present review.

An extensive literature search was performed, up to October 2017, on PubMed with the following combination of key words: “Epigenetics and stroke” and “EWAs and stroke”. For the combination of “Epigenetics and stroke” we found 128 papers and 4 for the combination of “EWAs and stroke”. Three papers were common between the two combinations of words (Fig. 1). Two researchers independently check the papers. We selected the papers that 1) were performed using human samples and an EWAs approach, and 2) papers that analysed the global methylation pattern of stroke patients. Finally, eight papers were included in the current revision (Fig. 1).

In addition, we included examples of other diseases or studies in animal models that support the results observed in stroke.

### 2.2 Stroke DNA Methylation Risk Factor

Epigenetic modifications, specifically DNA methylation, are influenced by environmental factors and are heritable modifications. It has been observed that different levels of DNA methylation are associated with the risk of diseases such as cancer, diabetes, obesity, atherosclerosis or arterial hypertension [35].

Human and mouse studies have observed global DNA hypermethylation of cytosines in CpGs as an accompanying feature of atherosclerosis [35], [36], [37]. Indeed, a positive correlation between DNA methylation and atherosclerotic lesion grade was discovered by using genome-wide DNA methylation sequencing (i.e., bisulphite sequencing) of healthy and atherosclerotic human aortas [35], [38] and confirmed by EWAS validation. Differentially methylated regions within the loci of cardiovascular disease associated genes in endothelial cells isolated from atherothrombotic regions of porcine aortas were also discovered using methylated DNA sequencing [39].

Furthermore, global DNA hypermethylation has been observed in peripheral lymphocytes of patients with cardiovascular disease, and may be linked to the inflammatory activity of innate immune cells [40].

Interestingly, global methylation was not found to be associated with stroke subtypes measured by luminometric methylation assay (LUMA) of blood DNA samples [41]. In contrast, other studies have observed an association of global hypomethylation and higher risk of stroke [42].

A recent study analysed a Swedish population of 729 subjects, 48 of them with myocardial infarction and 27 with previous stroke, using an EWAs approach. The authors found a different methylation pattern in 211 CpG sites associated with myocardial infarction. However, they did not find any significant association with stroke [43].

The DNA methylation levels change during aging, and there is usually a global hypermethylation in older people. In fact, is possible to calculate the biological age using the methylation levels of several CpG sites.

In the field of stroke, it has been shown that the biological age (different from chronological age) of stroke patients measured by DNA methylation is different between cases suffering stroke and healthy controls. The stroke patients were biologically older than healthy controls [44]. The authors of this study calculated the “epigenetic age” or “biological age” using the DNAm levels of whole-blood DNA and using the Hannum method [44], [45]. This method is based on the methylation levels from 71 methylation probes from a 450 K EWAS Methylation array.

The authors analysed a group of 123 stroke cases and controls matched by chronological age and they found that ischemic stroke patients were biologically on average 2.5 years older than healthy controls, indicating that biological age measured with DNA methylation levels could be a stronger risk factor for ischemic stroke than chronological age.

### 2.3 DNA Methylation and Stroke Recurrence

Several independent papers have observed the role of DNA methylation in the risk of new vascular events (vascular recurrence) after a first ischemic stroke. Hypomethylation of the *ABCB1* promoter has been associated with poor response to clopidogrel in Chinese ischemic stroke patients with the CYP2C19*1/*1 genotype [46]. ABCC3, another member of the ABC family, has been associated with the efflux of clopidogrel and its antiplatelet activity [47], [48]. However, *ABCC3* promoter methylation and down-regulation of *ABCC3* mRNA had no significant association with clopidogrel response [49].

A recent epigenome-wide study revealed that lower methylation of cg03548645 within the *TRAF3* body was associated with increased platelet aggregation and vascular recurrence in ischemic stroke patients treated with clopidogrel [50]. The authors hypothesised that higher *TRAF3* expression due to decreased methylation may lead to an increase in CD40 signal pathway interfered platelet-platelet interactions [51], [52].

In another study from the same group they observed that higher methylation levels of cg04985020 (*PPM1A* gene) were associated with vascular recurrence in patients treated with aspirin [53]. They analysed 38 ischemic stroke patients with an EWAS array and detected > 450,000 CpG sites. Secondly, they performed a replication analysis in 289 new ischemic stroke patients. This analysis confirmed that the role of *PPM1A* methylation levels in the risk of new vascular events after stroke involves the regulation of transforming growth factor. They hypothesised that higher methylation of *PPM1A* was associated with (TGF)-β1 signalling and plasminogen activator inhibitor-1 transcription [54].

### 2.4 DNA Methylation of Functional Outcome

In relation to functional outcome, only a couple of studies have been performed to evaluate the association of DNA methylation levels and post-stroke functional outcome. However, it seems that DNA methylation could have an important role in this condition. In studies with animal models of ischemia, it has been observed that epigenetic mechanisms are associated with neurological outcome, thereby indicating that epigenetic processes may play a significant role in post-stroke outcome [55].

Pilot studies with 700 stroke patients and EWAS data that analysed 450,000 CpG islands across the genome showed that several genes had an altered methylation associated with neurological outcome after stroke [56]. However, these results should be replicated in independent cohorts.

In addition, a recently published paper observed that biological age, as measured by DNA methylation, was associated with poor functional outcome in stroke patients [57]. The authors calculated the epigenetic age as the sum of the beta values multiplied by the reported effect sizes following a previously reported protocol [44], [45]. They observed that this epigenetic age was a better predictor of functional stroke outcome at the third month compared to chronological age. Interestingly, the same authors observed an association of epigenetic age with stroke risk [44].

### 2.5 Challenges and Solutions for Studying Epigenetics in Stroke Patients

Epigenetic analysis in stroke entails the challenge of testing the key tissue for the disease. It is difficult to study brain necrotic samples or human blood vessels; consequently, epigenetics studies are performed on blood samples. This could be a problem due to: a) the tissue not being the key tissue for the disease, and b) the variability in the cell composition of the blood samples.

For the first point, transcriptomic and proteomic analyses have demonstrated that blood is an important tissue for stroke, probably due to the role of leukocytes in the inflammation process of atherosclerosis and neurological outcome after stroke [58], [59].

For the second point, the epigenetic results can be normalised whenever the cell count data for a sample is available. In addition, for EWAS analysis, R packages (WateRmelon) [60], [61] incorporate statistical normalization for blood sample results.

## 3 Summary and Outlook

The use of epigenetics studies in stroke is an emerging field of interest. It seems that epigenetics plays an important role in this disease in different stages, prior to the ischemic attack and after the ischemic stroke. Specifically, DNA methylation could be associated with the risk of stroke, with stroke recurrence and with the functional outcome after stroke.

DNA methylation is a modifiable regulation; it is possible that in the future methylated or unmethylated genes could be a drug target for stroke treatment. In fact, there are now effective neuroprotective drugs that can be used to improve neurological worsening after stroke. New treatments focusing on this end-point could be very interesting for clinical practice.

However, more studies increasing the sample size with international collaboration and robust replications will be needed to find the epigenetic regulations that could be associated with stroke.

Several studies are currently ongoing in this field, mainly using EWAS arrays due to the option to perform genome-wide unbiased approaches. These studies will highlight the role of DNA methylation in stroke (Table 1).

**Table 1:** Summary of the most interesting epigenetics studies performed in stroke.

Author (reference)	Epigenetics association	Stroke risk	Stroke recurrence	Stroke functional outcome
Zaina et al. [35]	Positive correlation between DNA methylation and atherosclerotic lesion grade	Yes	na	na
Valencia-Morales et al. [38]	Correlation between histological grade of aortic stenosis and DNA methylation	Yes	na	na
Soriano et al. [41]	Global Methylation	No	na	na
Soriano et al. [44]	“Biological age” measured with DNA methylation	Yes	na	na
Yang et al. [46]	Hypomethylation of the *ABCB1* promoter has been associated with poor response to clopidogrel in Chinese ischemic stroke patients with the CYP2C19*1/*1 genotype	na	Yes	na
Jie et al. [49]	*ABCC3* promoter methylation and down-regulation of *ABCC3* mRNA had no significant association with clopidogrel response	na	No	na
Gallego-Fabrega et al. [50]	Lower methylation of cg03548645 within the *TRAF3* body was associated with increased platelet aggregation and vascular recurrence in ischemic stroke patients treated with clopidogrel	na	Yes	na
Gallego-Fabrega et al. [53]	Higher methylation levels of cg04985020 (*PPM1A* gene) were associated with vascular recurrence in patients treated with aspirin	na	Yes	na
Cullell et al. [56]	Pilot studies with 700 stroke patients and EWAS data that analysed 450,000 CpG islands across the genome, showed that several genes had an altered methylation associated with neurological outcome after stroke	na	na	Yes
Soriano-Tarrega et al. [57]	Biological age, as measured by DNA methylation, was associated with poor functional outcome in stroke patients	na	na	Yes
Rask-Andersen et al. [43]	EWAs performed in 729 subjects (strokes n = 27) did not find significant associations with stroke.	No	na	na

In addition, epigenetic treatment has been approved by regulatory agencies for several conditions. The relevance of epigenetic treatment in haematological malignancies (leukemia, lymphomas, myelodysplastic syndromes, myeloma) have already been described in detail [62]. Several agents that interfere with DNA methylation-demethylation and histones acetylation/deacetylation have been studied, and some (such as azacytidine, decitabine, valproic acid and vorinostat) are already in clinical use. In the current clinical setting, there are two classes of epigenetic drugs, which act through inhibition of the enzymatic activities responsible for epigenetic transcriptional silencing: DNMTs and HDACs. Importantly, one type of HDAC, HDAC9, has been associated with stroke risk in several genome wide studies [9], [13]. In addition, recent papers using HDACs inhibitors have been associated with better functional recovery in ischemic models of stroke [63]. This type of drugs could be tested in stroke in the future if epigenetic associations with stroke risk and stroke outcome are confirmed.

The authors of the review “*DNA methylation in ischemic stroke. Update of last advances.*” declare no disclosures and no conflict of interest.

## References

[1] Jorgensen N., Cabañas M., Oliva J., Rejas J., León T. (2008). The cost of informal care associated to incapacitating neurological disease having high prevalence in Spain. Neurologia.

[2] Di Carlo A., Launer L.J., Breteler M.M., Fratiglioni L., Lobo A. (2000). Frequency of stroke in Europe: a collaborative study of population-based cohorts ILSA working group and the neurologic diseases in the elderly research group Italian longitudinal study on aging. Neurology.

[3] Evers S.M., Struijs J.N., Ament A.J., van Genugten M.L., Jager J.H. (2004). International comparison of stroke cost studies. Stroke.

[4] Fernández-Cadenas I., Mendióroz M., Giralt D., Nafria C., Garcia E. (2017). GRECOS project (genotyping recurrence risk of stroke): the use of genetics to predict the vascular recurrence after stroke. Stroke.

[5] Roquer J., Ois A., Rodríguez-Campello A., Gomis M., Munteis E. (2007). Atherosclerotic burden and early mortality in acute ischemic stroke. Arch Neurol.

[6] Cuadrado-Godia E., Jiménez-Conde J., Ois A., Rodríguez-Campello A., García-Ramallo E. (2009). Sex differences in the prognostic value of the lipid profile after the first ischemic stroke. J Neurol.

[7] Heitsch L. (2015). International stroke conference.

[8] Holliday E.G., Maguire J.M., Evans T.J., Koblar S.A., Jannes J. (2012). Common variants at 6p21.1 are associated with large artery atherosclerotic stroke. Nat Genet.

[9] Traylor M., Farrall M., Holliday E.G., Sudlow C., Hopewell J.C. (2012). Genetic risk factors for ischaemic stroke and its subtypes (the METASTROKE collaboration): a meta-analysis of genome-wide association studies. Lancet Neurol.

[10] Bellenguez C., Bevan S., Gschwendtner A., Spencer C.C., Burgess A.I. (2012). Genome-wide association study identifies a variant in HDAC9 associated with large vessel ischemic stroke. Nat Genet.

[11] Gretarsdottir S., Thorleifsson G., Manolescu A., Styrkarsdottir U., Helgadottir A. (2008). Risk variants for atrial fibrillation on chromosome 4q25 associate with ischemic stroke. Ann Neurol.

[12] Gudbjartsson D.F., Holm H., Gretarsdottir S., Thorleifsson G., Walters G.B. (2009). A sequence variant in ZFHX3 on 16q22 associates with atrial fibrillation and ischemic stroke (2009). Nat Genet.

[13] Neurology Working Group of the Cohorts for Heart and Aging Research in Genomic Epidemiology (CHARGE) Consortium, Stroke Genetics Network (SiGN), International Stroke Genetics Consortium (ISGC) (2016). Identification of additional risk loci for stroke and small vessel disease: a meta-analysis of genome-wide association studies. Lancet Neurol.

[14] Traylor M., Mäkelä K.M., Kilarski L.L., Holliday E.G., Devan W.J. (2014). A novel MMP12 locus is associated with large artery atherosclerotic stroke using a genome-wide age-at-onset informed approach. PLoS Genet.

[15] Williams F.M., Carter A.M., Hysi P.G., Surdulescu G., Hodgkiss D. (2013). Ischemic stroke is associated with the ABO locus: the EuroCLOT study. Ann Neurol.

[16] Kubo M., Hata J., Ninomiya T., Matsuda K., Yonemoto K. (2007). A nonsynonymous SNP in PRKCH (protein kinase C eta) increases the risk of cerebral infarction. Nat Genet.

[17] Ikram M.A., Seshadri S., Bis J.C., Fornage M., DeStefano A.L. (2009). Genomewide association studies of stroke. N Engl J Med.

[18] Hsieh Y.C., Seshadri S., Chung W.T., Hsieh F.I., Hsu Y.H. (2012). Association between genetic variant on chromosome 12p13 and stroke survival and recurrence: a one year prospective study in Taiwan. J Biomed Sci.

[19] Zhang W., Chen Y., Liu P., Chen J., Song L. (2012). Variants on chromosome 9p21.3 correlated with ANRIL expression contribute to stroke risk and recurrence in a large prospective stroke population. Stroke.

[20] Williams S.R., Hsu F.C., Keene K.L., Chen W.M., Nelson S. (2016). Shared genetic susceptibility of vascular-related biomarkers with ischemic and recurrent stroke. Neurology.

[21] Achterberg S., Kappelle L.J., de Bakker P.I., Traylor M., Algra A., SMART Study Group and the METASTROKE Consortium (2015). No additional prognostic value of genetic information in the prediction of vascular events after cerebral ischemia of arterial origin: the PROMISe study. PLoS One.

[22] Manso H., Krug T., Sobral J., Albergaria I., Gaspar G. (2010). Variants of the matrix Metalloproteinase-2 but not the matrix metalloproteinase-9 genes significantly influence functional outcome after stroke. BMC Med Genet.

[23] Maguire J., Thakkinstian A., Levi C., Lincz L., Bisset L. (2011). Impact of COX-2 rs5275 and rs20417 and GPIIIa rs5918 polymorphisms on 90-day ischemic stroke functional outcome: a novel finding. J Stroke Cerebrovasc Dis.

[24] Gomez-Sanchez J.C., Delgado-Esteban M., Rodriguez-Hernandez I., Sobrino T., Perez de la Ossa N. (2011). The human Tp53 Arg72Pro polymorphism explains different functional prognosis in stroke. J Exp Med.

[25] Bevan S., Traylor M., Adib-Samii P., Malik R., Paul N.L. (Dec 2012). Genetic heritability of ischemic stroke and the contribution of previously reported candidate gene and genomewide associations. Stroke.

[26] Domingues-Montanari S., Mendioroz M., del Rio-Espinola A., Fernández-Cadenas I., Montaner J. (2008). Genetics of stroke: a review of recent advances. Expert Rev Mol Diagn.

[27] Henikoff S., Matzke M.A. (1997). Exploring and explaining epigenetic effects. Trends Genet.

[28] Takai D., Jones P.A. (2002). Comprehensive analysis of CpG islands in human chromosomes 21 and 22. Proc Natl Acad Sci U S A.

[29] Shahbazian M.D., Grunstein M. (2007). Functions of site-specific histone acetylation and deacetylation. Annu Rev Biochem.

[30] Greißel A., Culmes M., Burgkart R., Zimmermann A., Eckstein H.H. (2016). Histone acetylation and methylation significantly change with severity of atherosclerosis in human carotid plaques. Cardiovasc Pathol.

[31] Jonas S., Izaurralde E. (2015). Towards a molecular understanding of microRNA-mediated gene silencing. Nat Rev Genet.

[32] Zorio E., Medina P., Rueda J., Millán J.M., Arnau M.A. (2009). Insights into the role of microRNAs in cardiac diseases: from biological signalling to therapeutic targets. Cardiovasc Hematol Agents Med Chem.

[33] Cancer Genome Atlas Research Network, Ley T.J., Miller C., Ding L., Raphael B.J., Mungall A.J. (2013). Genomic and epigenomic landscapes of adult de novo acute myeloid leukemia. N Engl J Med.

[34] Khyzha N., Alizada A., Wilson M.D., Fish J.E. (2017 Apr). Epigenetics of atherosclerosis: emerging mechanisms and methods. Trends Mol Med.

[35] Zaina S., Heyn H., Carmona F.J., Varol N., Sayols S. (2014). DNA methylation map of human atherosclerosis. Circ Cardiovasc Genet.

[36] Yoo T., Yoo T., Yoon Y.S., Ryu S.H., Ahn J.Y. (2012). Hypermethylation of repetitive DNA elements in livers of mice fed an atherogenic diet. Nutrition.

[37] Rangel-Salazar R. (2011). Human native lipoprotein-induced de novo DNA methylation is associated with repression of inflammatory genes in THP-1 macrophages. BMC Genomics.

[38] Valencia-Morales Mdel P., Zaina S., Heyn H., Carmona F.J., Varol N. (2015). The DNA methylation drift of the atherosclerotic aorta increases with lesion progression. BMC Med Genomics.

[39] Jiang Y.Z., Jiménez J.M., Ou K., ME McCormick, Zhang L.D. (2014). Hemodynamic disturbed flow induces differential DNA methylation of endothelial Kruppel-like factor 4 promoter in vitro and in vivo. Circ Res.

[40] Sharma P., Kumar J., Garg G., Kumar A., Patowary A. (2008). Detection of altered global DNA methylation in coronary artery disease patients. DNA Cell Biol.

[41] Soriano-Tárraga C., Jiménez-Conde J., Giralt-Steinhauer E., Mola M., Ois A. (2014). Global DNA methylation of ischemic stroke subtypes. PLoS One.

[42] Baccarelli A., Wright R., Bollati V., Litonjua A., Zanobetti A. (2010). Ischemic heart disease and stroke in relation to blood DNA methylation. Epidemiology.

[43] Rask-Andersen M., Martinsson D., Ahsan M., Enroth S., Ek W.E. (2016). Epigenome-wide association study reveals differential DNA methylation in individuals with a history of myocardial infarction. Hum Mol Genet.

[44] Soriano-Tárraga C., Giralt-Steinhauer E., Mola-Caminal M., Vivanco-Hidalgo R.M., Ois A. (2016). Ischemic stroke patients are biologically older than their chronological age. Aging (Albany NY).

[45] Hannum G., Guinney J., Zhao L., Zhang L., Hughes G. (2013). Genome-wide methylation profiles reveal quantitative views of human aging rates. Mol Cell.

[46] Yang J., Zhou J.S., Zhao Y.X., Yang Z.H., Zhao (2015). ABCB1 hypomethylation is associated with decreased antiplatelet effects of clopidogrel in Chinese ischemic stroke patients. Pharmazie.

[47] Luchessi A.D., Silbiger V.N., Cerda A., Hirata R.D., Carracedo A. (2012). Increased clopidogrel response is associated with ABCC3 expression: a pilot study. Clin Chim Acta.

[48] Zou J.J., Fan H.W., Chen S.L., Tan J., He B.S. (2013). Efffect of the ABCC3-211C/T polymorphism on clopidogrel responsiveness in patients with percutaneous coronary intervention. Clin Exp Pharmacol Physiol.

[49] Jie Y., Jun-Shan Z., Ying-Dong Z., You-Yong T., Jian-Jun Z. (2014). The association of ABCC3 promoter methylation with clopidogrel response in Chinese ischemic stroke patients. Pharmazie.

[50] Gallego-Fabrega C., Carrera C., Reny J.L., Fontana P., Slowik A. (2016). TRAF3 epigenetic regulation is associated with vascular recurrence in patients with ischemic stroke. Stroke.

[51] Song Z., Jin R., Yu S., Rivet J.J., Smyth S.S. (2011). CD40 is essential in the upregulation of TRAF proteins and NF-kappaB-dependent proinflammatory gene expression after arterial injury. PLoS One.

[52] Kuijpers M.J., Mattheij N.J., Cipolla L., van Geffen J.P., Lawrence (2015). Platelet CD40L modulates thrombus growth via phosphatidylinositol 3-kinase beta, and not via CD40 and IkappaB kinase alpha. Arterioscler Thromb Vasc Biol.

[53] Gallego-Fabrega C., Carrera C., Reny J.L., Fontana P., Slowik A. (2016). PPM1A methylation is associated with vascular recurrence in aspirin-treated patients. Stroke.

[54] Samarakoon R., Chitnis S.S., Higgins S.P., Higgins C.E., Krepinsky J.C. (2011). Redox-induced Src kinase and caveolin-1 signaling in TGF- β1-initiated SMAD2/3 activation and PAI-1 expression. PLoS One.

[55] Kassis H., Shehadah A., Li C., Zhang Y., Cui Y. (2016). Class IIa histone deacetylases affect neuronal remodeling and functional outcome after stroke. Neurochem Int.

[56] Woo D., Debette S., Anderson C. (2017 Mar 30). 20th Workshop of the International Stroke Genetics Consortium, November 3-4, 2016, Milan, Italy: 2016.036 ISGC research priorities. Neurol Genet.

[57] Soriano-Tárraga C., Mola-Caminal M., Giralt-Steinhauer E., Ois A., Rodríguez-Campello A. (2017). Biological age is better than chronological as predictor of 3-month outcome in ischemic stroke. Neurology.

[58] Cox C., Sharp F.R. (2013). RNA-based blood genomics as an investigative tool and prospective biomarker for ischemic stroke. Neurol Res.

[59] García-Berrocoso T., Penalba A., Boada C., Giralt D., Cuadrado E. (2013). From brain to blood: new biomarkers for ischemic stroke prognosis. J Proteomics.

[60] Touleimat N., Tost J. (2012). Complete pipeline for Infinium®, human methylation 450K BeadChip data processing using subset quantile normalization for accurate DNA methylation estimation. Epigenomics.

[61] Pidsley R.Y., Wong C.C., Volta M., Lunnon K., Mill J. (2013). A data-driven approach to preprocessing Illumina 450K methylation array data. BMC Genomics.

[62] Santini V., Melnick A., Maciejewski J.P., Duprez E., Nervi C. (2013). Epigenetics in focus: pathogenesis of myelodysplastic syndromes and the role of hypomethylating agents. Crit Rev Oncol Hematol.

[63] Tang Y., Lin Y.H., Ni H.Y., Dong J., Yuan H.J. (2017). Inhibiting histone deacetylase 2 (HDAC2) promotes functional recovery from stroke. J Am Heart Assoc.

